# IL-6, IL-8, and IL-10 Are Associated with Hyperferritinemia in Rapidly Progressive Interstitial Lung Disease with Polymyositis/Dermatomyositis

**DOI:** 10.1155/2014/815245

**Published:** 2014-04-01

**Authors:** Hidenaga Kawasumi, Takahisa Gono, Yasushi Kawaguchi, Hirotaka Kaneko, Yasuhiro Katsumata, Masanori Hanaoka, Sayuri Kataoka, Hisashi Yamanaka

**Affiliations:** Institute of Rheumatology, Tokyo Women's Medical University, 10-22 Kawada-Cho, Shinjuku-Ku, Tokyo 162-0054, Japan

## Abstract

*Objective.* Hyperferritinemia is frequently accompanied by rapidly progressive (RP) interstitial lung disease (ILD) with polymyositis (PM)/dermatomyositis (DM). To clarify the mechanism of RP-ILD with hyperferritinemia, we investigated the associations between serum ferritin levels and various cytokines in patients with PM/DM. *Methods.* This retrospective study included 38 patients admitted to our hospital with PM/DM. Levels of serum ferritin and cytokines (IL-1**β**, IL-2, IL-4, IL-6, IL-8, IL-10, IL-12, IL-13, IL-17, IL-18, TNF-**α**, IFN-**α**, IFN-**γ**, and IP-10) were measured. Disease activity was evaluated using the tool proposed by the International Myositis Assessment and Clinical Studies Group. We analyzed the associations between disease activity and levels of serum ferritin and cytokines. *Results.* The levels of serum ferritin, IL-8, IL-10, IL-18, and TNF-**α**, were significantly correlated with disease activity. In a multivariate analysis, IL-6 (*t* = 3.6, *P* = 0.0010), IL-8 (*t* = 4.8, *P* < 0.0001), and IL-10 (*t* = 5.7, *P* < 0.0001) significantly contributed to serum ferritin levels. The levels of serum ferritin, IL-6, IL-8, and IL-10, were higher in the RP-ILD subset than in the non-ILD subset or the chronic ILD subset. *Conclusion.* IL-6, IL-8, and IL-10 are significant contributors to hyperferritinemia in PM/DM. The regulation of these cytokines might offer a possible treatment strategy for RP-ILD with PM/DM.

## 1. Introduction

Polymyositis (PM) and dermatomyositis (DM) are idiopathic inflammatory myopathies that are characterized by inflammation of the muscles, skin, lungs, and heart. PM/DM is occasionally complicated by interstitial lung disease (ILD) [1]. ILD is a prognostic factor for PM/DM [2, 3]. In particular, rapidly progressive (RP) ILD is an intractable and life-threatening complication in PM/DM. In Japan, the overall 6-month survival rate following diagnosis is approximately 50–60% in RP-ILD associated with antimelanoma differentiation-associated gene 5 (MDA-5) antibody [4, 5]. The anti-MDA-5 antibody is detected in half of clinically amyopathic DM (CADM) patients [5]. Measuring myositis-specific autoantibodies is useful for predicting clinical manifestations, the response to treatment, and prognosis [3, 6]. Among anti-MDA-5 associated ILDs, RP-ILD is frequently observed in East Asia, although chronic ILD is usually found in western countries [7]. Anti-aminoacyl-tRNA synthetase (ARS) antibody, including anti-Jo-1, is also associated with complications of ILD that generally lead to a progressively chronic condition, although they are occasionally also associated with rapid progression in PM/DM [1].

We previously reported that high levels of serum ferritin are associated with the severity and prognosis of ILD with PM/DM [8]. Hyperferritinemia could be associated with a flood of cytokines due to macrophage activation in PM/DM as well as macrophage activation syndrome (MAS)/hemophagocytic syndrome (HPS). In addition, we previously demonstrated that accumulated macrophages synthesized ferritin in the lung and bone marrow of a patient with RP-ILD associated with DM [9]. However, the mechanism of hyperferritinemia in RP-ILD with PM/DM has remained unclear.

Therefore, we investigated the associations between levels of serum ferritin and several cytokines and clarified the mechanism of hyperferritinemia during pulmonary disease activity in PM/DM.

## 2. Patients and Methods

### 2.1. Patients

In this study, patients who were admitted to Tokyo Women's Medical University Aoyama Hospital from 1992 to 2010 were retrospectively examined. Diagnoses of PM/DM or CADM were made based on the criteria of Bohan and Peter [10] or Sontheimer [11], respectively. Specific rashes, including heliotrope rash, Gottron's sign, or Gottron's papules, were used to identify DM.

Within this cohort, we found that one hundred and twenty-four patients had been diagnosed with PM, DM, or CADM. Of these 124 patients, 78 and 46 patients were complicated with and without ILD, respectively. Among these 124 patients, sera could be obtained from 38 patients (21 with ILD and 17 without ILD) during the active phase. All of the 38 enrolled patients suffered from skin rashes, myopathy, or respiratory symptoms (or a combination thereof). For all enrolled patients, the disease status was active upon admission, leading the physicians to begin immunosuppressive therapy or increase the intensity of therapy. The disease duration was defined as the time between the appearance of PM/DM-associated symptoms and admission. The clinical data were obtained from medical records when the serum samples were obtained. This study was approved by the Ethical Committee of Tokyo Women's Medical University according to the Declaration of Helsinki.

### 2.2. Classification of ILD

Pulmonary disease was assessed using chest radiography and computed tomography. RP-ILD was defined as a progressive dyspnea that developed rapidly, over days to weeks, after the onset of respiratory symptoms. Chronic ILD was defined as a progressive dyspnea that developed slowly, over months [4, 5].

### 2.3. Evaluation of Myositis-Specific Autoantibodies

Anti-MDA-5 Ab was detected using an enzyme-linked immunosorbent assay with recombinant MDA-5 as an antigen, as described previously [12]. Anti-ARS Abs, including Jo-1, EJ, PL-7, PL-12, and OJ, were measured using RNA immunoprecipitation assays. The other myositis-specific autoantibodies (MSAs) were detected through immunoblots using the Myositis Profile Euroline antibody test system for the in vitro determination of antibodies against myositis-associated antigens in human serum (Euroimmun, Lübeck, Germany).

### 2.4. Evaluation of Disease Activity for PM/DM/CADM

Disease activity was evaluated using the myositis disease activity core set proposed and recommended by the IMACS [13]. The activities of global disease and ILD were measured using the visual analogue scale (VAS) by physicians. Disease activity is defined as a potentially reversible finding resulting from PM/DM. Physicians rate overall assessment of the ongoing activity for the global disease and the pulmonary disease on the 0–10 cm VAS scale by drawing a vertical mark on the 10 cm line according to the following scale: left end of line = no evidence of disease activity, midpoint of line = moderate disease activity, and right end of line = extremely active or severe disease activity. The global VAS and pulmonary VAS were estimated retrospectively by physicians in charge or evaluators who were blinded to the results of ferritin and cytokine profiles in reference to the medical record information.

### 2.5. Measurement of Cytokines and Ferritin

Serum samples were stored at −80°C. The levels of serum cytokines, including IL-1*β*, IL-2, IL-4, IL-6, IL-8, IL-10, IL-12, IL-13, IL-17, TNF-*α*, IFN-*α*, IFN-*γ*, and interferon *γ*-inducible 10-kd (IP-10), were measured using a multiplex assay with the Milliplex MAP Human Cytokine/Chemokine Panel (EMD Millipore Corp., MA, USA), Bio-Plex 200 system, and Bio-Plex Manager software version 6.0 (Bio-Rad Laboratories, Inc., CA, USA). Serum IL-18 was measured using an enzyme-linked immunosorbent assay (R & D Systems, Minneapolis, MN). Ferritin was measured using a chemiluminescent immunoassay.

### 2.6. Statistical Analysis

Statistical analyses were performed using the Mann-Whitney *U*-test for comparisons of median values. Correlation coefficients were established by employing Spearman's rank correlation coefficients. Multiple linear regression analysis was performed when appropriate. The data were analyzed using JMP software (SAS Institute, NC, USA). A *P* value <0.05 indicated statistical significance.

## 3. Results

### 3.1. Clinical Characteristics of the Enrolled Patients

As shown in Table 1, 38 patients were enrolled in this study. The median age was 52 years, and 74% of the participants were females. The numbers of PM, DM, and CADM cases were 12, 14, and 12, respectively. The median disease duration was 4 months. The frequency of ILD was 55%. In addition, the frequencies of RP-ILD and chronic ILD were 26% and 29%. The autoantibody-positivity rates of anti-ARS, anti-Mi-2, anti-MDA-5, and anti-SRP were 32%, 3%, 26%, and 5%, respectively. Among the 10 patients with RP-ILD, anti-MDA5 and anti-ARS were detected in 8 and 2 patients. However, among the 11 patients with chronic ILD, anti-MDA5 and anti-ARS were detected in 2 and 9 patients.

### 3.2. Correlation between Disease Activity and Cytokine Levels

We evaluated the association between the disease activity (global VAS and pulmonary VAS) and the levels of each cytokine. As shown in Table 2, the global VAS was significantly correlated with the levels of IL-6 (*r*_*s*_ = 0.47), IL-8 (*r*_*s*_ = 0.51), IL-10 (*r*_*s*_ = 0.50), IL-18 (*r*_*s*_ = 0.53), TNF-*α* (*r*_*s*_ = 0.39), and IP-10 (*r*_*s*_ = 0.44). In addition, the pulmonary VAS was significantly correlated with the levels of IL-8 (*r*_*s*_ = 0.45), IL-10 (*r*_*s*_ = 0.46), IL-18 (*r*_*s*_ = 0.34), TNF-*α* (*r*_*s*_ = 0.45), and IP-10 (*r*_*s*_ = 0.33).

Serum ferritin levels were also significantly correlated with the global VAS (*r*_*s*_ = 0.50) and the pulmonary VAS (*r*_*s*_ = 0.62).

### 3.3. Correlation between Serum Ferritin and Cytokine Levels

We evaluated the association between ferritin levels and the levels of each cytokine. As shown in Table 3, serum ferritin levels were significantly correlated with the levels of IL-6 (*r*_*s*_ = 0.40), IL-8 (*r*_*s*_ = 0.57), IL-10 (*r*_*s*_ = 0.50), IL-18 (*r*_*s*_ = 0.46), and TNF-*α* (*r*_*s*_ = 0.34).

To clarify the degree to which these cytokines contribute to the levels of serum ferritin, we performed a multiple linear regression analysis. IL-6 (*t* = 2.9, *P* < 0.05), IL-8 (*t* = 3.6, *P* < 0.01), and IL-10 (*t* = 4.0, *P* < 0.001) were significantly associated with serum ferritin levels.

Previously, we confirmed that hyperferritinemia, which was defined as greater than 500 ng/mL in MAS/HPS, predicted the development of RP-ILD in PM/DM [8]. Therefore, cut-off values of IL-6, IL-8, and IL-10 for hyperferritinemia were calculated from the receiver operating characteristic curve. The results were as follows: IL-6, 2.41 pg/mL; IL-8, 15.73 pg/mL; and IL-10, 11.51 pg/mL.

### 3.4. Comparison of Serum Ferritin or Cytokine Levels among PM/DM in the Non-ILD Subset, the Chronic ILD Subset, and the RP-ILD Subset

We divided the 38 enrolled patients into three subsets: a non-complication of ILD (non-ILD) subset (*n* = 17), a chronic ILD subset (*n* = 11), and a RP-ILD subset (*n* = 10). We compared the levels of serum ferritin and each cytokine among the three subsets.

**Figure 1:**
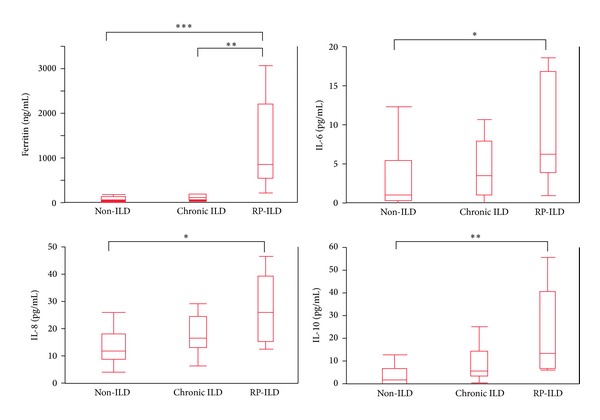
Comparison of the levels of serum ferritin and cytokines in the non-ILD subset, the chronic ILD subset, and the RP-ILD subset. We divided the 38 enrolled patients into three subsets: a non-complication of ILD (non-ILD) subset (*n* = 17), a chronic ILD subset (*n* = 11), and a RP-ILD subset (*n* = 10). Statistical analyses were performed using the Mann-Whitney *U*-test for comparisons of median values. *P* values are as indicated below. * < 0.05, ** < 0.01, and *** < 0.001. The median levels (ranges) of IL-6, IL-8, and IL-10 were 0 pg/mL (0–1.3), 1.7 pg/mL (0.9–6.0), and 0 pg/mL (0-0), respectively, in the 15 healthy controls.

As shown in Figure 1, serum ferritin levels were significantly higher in the RP-ILD subset than in the non-ILD subset (*P* = 0.0005) or chronic ILD subset (*P* = 0.001). IL-6, IL-8, and IL-10 levels were higher in the RP-ILD subset than in the non-ILD subset (*P* = 0.020, 0.017, and 0.003, resp.) or chronic ILD subset (*P* = 0.19, 0.072, and 0.057, resp.), although the differences were not statistically significant in the chronic ILD subset.

## 4. Discussion

We demonstrated associations between disease activity, several cytokines, and serum ferritin levels in PM/DM. IL-6, IL-8, and TNF-*α* were previously correlated with global disease activity in PM/DM [14], consistent with the results of the present study. However, the associations between pulmonary disease activity and several cytokines have remained unclear. The present study revealed that IL-8, IL-10, IL-18, TNF-*α*, and IP-10 are significantly correlated with pulmonary disease activity and global disease activity. When considering treatment for ILD with PM/DM, the regulation of these inflammatory cytokines could be critical.

Serum ferritin levels were significantly correlated with pulmonary disease activity in the present study. Consequently, serum ferritin could be a biomarker for evaluating pulmonary disease activity. We previously reported that serum ferritin levels were higher in RP-ILD with DM than in chronic ILD with DM or non-ILD with DM [8]. Moreover, the present study demonstrated that there was no significant difference between 9 non-ILD patients with PM and 8 non-ILD patients with DM in the serum ferritin, IL-6, IL-8, and IL-10 levels (data not shown). Thus, we consider that hyperferritinemia and this characteristic profile of cytokines specifically result from pathophysiology of RP-ILD with PM/DM, although the precise mechanism of hyperferritinemia in RP-ILD with PM/DM has remained unclear. Hyperferritinemia is apparent in MAS/HPS. The pathophysiology of MAS/HPS involves the activation of macrophages and T_H_1 cells and the excessive production of cytokines, such as IL-1*β*, IL-6, IL-8, IL-10, IL-18, TNF-*α*, and IFN-*γ*. In RP-ILD associated with anti-MDA-5 positive CADM, hyperferritinemia is frequently observed [4, 5]. Previously, we demonstrated the presence of systemic ferritin producing macrophages in DM with RP-ILD and hyperferritinemia [9]. The accumulated cells detected in the alveoli were predominantly CD68 positive and synthesis ferritin. These CD68 positive cells were observed diffusely in the bone marrow, liver, and spleen. Moreover, ferritin is a reliable specific marker for macrophages [15]. Taken together, we consider that systemic macrophages are activated in PM/DM patients with RP-ILD. In the present study, serum ferritin levels were significantly correlated with the levels of IL-6, IL-8, IL-10, IL-18, and TNF-*α*. The pathophysiology of RP-ILD with PM/DM could be similar to that of MAS/HPS.

Ferritin, which is synthesized mainly by macrophages, is the major intracellular protein responsible for the storage of iron. Proinflammatory cytokines, including IL-1*β*, IL-6, and TNF-*α*, and the anti-inflammatory cytokine IL-10 directly stimulate the transcription and translation of ferritin [16]. The synthesis of IL-10 is induced by these proinflammatory cytokines. IL-1*β* and IL-6 also induce the synthesis of hepcidin in the liver. Hepcidin is released into the circulation and binds the cellular iron export protein ferroportin-1. Hepcidin degrades ferroportin-1 expressed on the outer cell membrane of macrophages, which leads to a reduction of macrophage iron export [17]. IL-10 and IFN-*γ* also stimulate iron uptake into macrophages via transferrin receptors or via divalent metal transporter-1 [17], resulting in iron retention and storage in macrophages, which manifests as hyperferritinemia. In the present study, serum ferritin levels were significantly correlated with the levels of IL-6, IL-8, and IL-10, which were higher in the RP-ILD subset than in the chronic ILD or non-ILD subsets of PM/DM. Thus, these cytokines are key mediators for the development of hyperferritinemia complicated with RP-ILD in PM/DM.

In general, activated macrophages are classified into three subsets: classically activated macrophages, wound-healing macrophages, and regulatory macrophages [18]. Classically activated macrophages produce proinflammatory cytokines, such as IL-1 and IL-6. Classically activated macrophages are stimulated by T_H_1. Plasma IL-8 levels have been associated with the severity of acute lung injury (ALI)/adult respiratory distress syndrome (ARDS) [19]. The IL-8 related neutrophil elastase may play a role in the pathogenesis of ALI/ARDS. In RP-ILD with PM/DM, classically activated macrophages may produce proinflammatory cytokines such as IL-6 and IL-8, stimulate neutrophils and lymphocytes, and cause injury in alveolar epithelial cells, such as diffuse alveolar damage.

There are several limitations to this study. Firstly, this study was retrospectively conducted. The global VAS and pulmonary VAS were retrospectively estimated. Secondly, in the present study, patient selection bias may be found because all patients were not consecutively enrolled. Some results might not be enough valid in the whole aspect of PM/DM-ILD. Finally, some patients were being treated with intermediate or high-dose PSL at the time of serum collection. These medications could influence the measurement of cytokine levels, although almost all patients were being treated with low-dose PSL (0.1-0.2 mg/kg/day) or with symptomatic therapy when serum was collected.

In conclusion, serum ferritin levels are correlated with pulmonary disease activity in PM/DM. IL-6, IL-8, and IL-10 are significant factors that contribute to serum ferritin levels. The regulation of these cytokines might be a possible treatment strategy for RP-ILD with PM/DM.

## Figures and Tables

**Table 1:** Clinical characteristics in the enrolled patients*.

	(*n* = 38)
Age, years	52 (39–58)
Female, number (%)	28 (74)
PM, DM, and CADM, number	12, 14, 12
Disease duration, month	4 (2–7)
Interstitial lung disease, number (%)	21 (55)
Rapidly progressive, number (%)	10 (26)
Chronic, number (%)	11 (29)
Myositis-specific autoantibodies, number (%)	
Anti-ARS	12 (32)
Anti-Mi-2	1 (3)
Anti-MDA-5	10 (26)
Anti-SRP	2 (5)

*Thirty-eight patients were enrolled in the present study.

Values are median (interquartile range).

PM: polymyositis; DM: dermatomyositis; CADM: clinically amyopathic DM; ARS: aminoacyl-tRNA synthetase; MDA-5: melanoma differentiation-associated gene 5; and SRP: signal recognition particle.

**Table 2:** The associations between disease activity, serum cytokine levels, and ferritin levels.

	Global VAS, *r*_*s*_	Pulmonary VAS, *r*_*s*_
IL-1*β*	−0.09	−0.03
IL-2	−0.01	0.06
IL-4	−0.04	0.31
IL-6	0.47**	0.33
IL-8	0.51**	0.45**
IL-10	0.50**	0.46**
IL-12	0.05	0.11
IL-13	−0.05	−0.01
IL-17	0.17	−0.05
IL-18	0.53**	0.34*
TNF-*α*	0.39*	0.45**
IFN-*α*	−0.03	−0.03
IFN-*γ*	0.20	0.11
IP-10	0.44*	0.33*
Ferritin	0.50*	0.62***

Statistical analyses were performed using Spearman's rank correlation coefficient.

*P* values are as indicated below. *P < 0.05,  **P < 0.01, and ***P < 0.001.

VAS: visual analogue scale and IP-10: interferon *γ*-inducible 10-kd protein.

**Table 3:** The associations between serum ferritin levels and cytokine levels.

	Ferritin, *r*_*s*_
IL-1*β*	0.00
IL-2	0.08
IL-4	−0.11
IL-6	0.40*
IL-8	0.57***
IL-10	0.50**
IL-12	0.10
IL-13	0.11
IL-17	0.23
IL-18	0.46**
TNF-*α*	0.34*
IFN-*α*	0.04
IFN-*γ*	0.24
IP-10	0.28

Statistical analyses were performed using Spearman's rank correlation coefficient.

*P* values are as indicated below. *P < 0.05, **P < 0.01, and ***P < 0.001.

VAS: visual analogue scale and IP-10: interferon *γ*-inducible 10-kd protein.
